# The Physical Fidelity Gap as an Evidence-Traceability Problem in AI Uncertainty Quantification: A Structured Review

**DOI:** 10.3390/s26175447

**Published:** 2026-08-28

**Authors:** Lin Guo, Aiwen Ma, Heng Zhou, Zilong Liu, Xingchuang Xiong

**Affiliations:** 1 Center for Metrology Scientific Data, National Institute of Metrology, Beijing 100029, China; guolin@tju.edu.cn (L.G.); zhouheng@nim.ac.cn (H.Z.); liuzl@nim.ac.cn (Z.L.); 2 National Metrology Data Center, Beijing 100029, China; 3 Key Laboratory of Metrology Digitalization and Digital Metrology, State Administration for Market Regulation, Beijing 100029, China; 4 Chinese Society for Measurement, Beijing 100025, China; maaiwen@sina.com

**Keywords:** AI uncertainty quantification, evidence traceability, physical fidelity gap, structured coding review, uncertainty claims, sensing and measurement, non-stationarity

## Abstract

**Highlights:**

**What are the main findings?**

- A total of 566 studies were included, of which 556 formed stable dominant AI uncertainty claim units and constituted the common analytic set for 15-dimensional coding.
- By evidence-traceability tier, 347 studies (62.4%) were classified as Weak, 133 (23.9%) as Medium, and 76 (13.7%) as Strong.

**What are the implications of the main findings?**

- Specific risk claims, Multiple uncertainty entry points, Multiple physical information types, and Sampling/ensemble approximation more often co-occurred with higher evidence traceability.
- PFG provides a scope-bounded reference for locating breakpoints in the claim–evidence chain and indicating directions for evidence strengthening; its interpretation is limited to the traceability of evidence publicly reported in the literature.

**Abstract:**

Artificial intelligence (AI) increasingly produces uncertainty outputs for sensing and measurement tasks, but the evidence supporting these outputs may not maintain a traceable correspondence with the relevant real-world conditions. This study conducted a structured 15-dimensional coding review of 566 studies, of which 556 formed stable dominant AI uncertainty claim units and entered the common analytic set. Each final code was linked to row-level audit evidence. The unidimensional distributions first showed breakpoint states with nonzero frequencies at the relevant nodes of the claim–condition–test–uncertainty-response chain, thereby confirming observable evidence discontinuities in the current corpus. The studies were then stratified using three sequential, non-compensatory evidence questions. Among the 556 studies, 347 (62.4%) were classified as Weak, 133 (23.9%) as Medium, and 76 (13.7%) as Strong. Descriptive cross-dimensional comparisons showed that specific OOD/drift risk or Error/quality estimation claims, Multiple uncertainty entry points, Multiple physical information types, and Sampling/ensemble approximation more often co-occurred with higher evidence traceability; Prediction reliability/confidence claims, a standalone Uncertainty proxy/score, and Latency/real-time inference constraints more often co-occurred with lower evidence traceability. This study summarizes the above evidence discontinuity as the physical fidelity gap (PFG), which refers to incomplete or unverifiable evidential links between AI uncertainty claims publicly reported in the literature and the relevant physical, measurement, or operational conditions. PFG provides a scope-bounded reference for locating links in the evidence chain that may need strengthening. The breakpoints observable in the current corpus indicate that the public evidence still has room for improvement in forming continuous, verifiable claim–condition–test–response correspondences; the tiered comparison indicates that subsequent work can strengthen the evidence chain by clarifying claim conditions, incorporating the relevant conditions into empirical testing, and reporting identifiable uncertainty responses and extended corroboration.

## 1. Introduction

AI uncertainty quantification has expanded from a problem concerning a single algorithm to an output problem in sensing, measurement, and monitoring chains; in addition to point estimates, models can provide predictive probabilities that require calibration [1], prediction intervals or sets [2], uncertainty scores such as variance and entropy [3], evidence measures [4], and posterior or predictive distributions [5]. These outputs may relate to data noise, insufficient model knowledge, prediction reliability, calibration status, or decision risk [6]. Around these objects, Bayesian neural networks [7], Monte Carlo dropout [8], deep ensembles [9], evidential deep learning [10], conformal prediction [11], post-hoc calibration [12], and out-of-distribution detection [13] have formed parallel families of methods. Different methods differ markedly in their applicable settings, computational costs, and empirical evaluation methods; method selection therefore still needs to account for system use and validation objectives [14]. The relevant outputs have been used in structural crack detection [15], ultrasonic crack characterization [16], precipitation nowcasting [17], agricultural decision support [18], medical image reconstruction [19], wireless-channel monitoring [20], machinery fault diagnosis [21], nonlinear dynamic measurement [22], and machine-vision measurement [23]. The coexistence of these methods, outputs, and uses also means that quantities termed “uncertainty” in the literature do not inherently have the same statistical meaning, real-world referent, or evidence requirements.

Existing evaluations mostly organize evidence by method, metric, or application: classification studies, including class-imbalanced settings, use confidence calibration [24] and reliability diagrams [25]; regression studies report coverage, interval width, negative log-likelihood, or probabilistic scores [26] and compare uncertainty from machine-learning and nonlinear least-squares models [27]; deployment studies examine dataset shift [28] or cross-domain semantic segmentation [29]. In reviews of engineering UQ, Wang et al. systematically examined probabilistic, non-probabilistic, and hybrid models, propagation algorithms, and surrogate modeling [30]; they subsequently organized uncertainty-based multidisciplinary design optimization into technical routes such as surrogate models, decomposition, and intelligent optimization [31]. In information fusion research, Pavlin et al. linked evaluation criteria for uncertainty representation and reasoning to the object being evaluated and to the system development–deployment life cycle [32]; Flores et al. further used the URREF representation and reasoning criteria to formally compare different uncertainty-handling approaches [33]. In metrology, the GUM provides systematic specifications for expressing measurement results and their uncertainty [34], and Carratù et al. also discussed the propagation of input measurement uncertainty through artificial neural networks [35]. However, most current AI UQ outputs are not equivalent to evaluations of measurement uncertainty in the metrological sense; probability, interval, or score outputs should therefore not be treated as metrological uncertainty evaluations without additional evidence.

This study focuses on a more fundamental problem currently present in AI UQ work: whether the claims supported by uncertainty outputs can form continuous, verifiable correspondences with real-world information, conditions of validity, empirical tests, uncertainty responses, and external corroboration. This study included 566 studies, of which 556 allowed stable identification of one dominant AI uncertainty claim unit and constituted a common analytic set. Uniform fields were then used to decompose the uncertainty outputs, claim objects, use contexts, real-world information and conditions, validation processes, uncertainty manifestations, and engineering and measurement interpretations reported by each study, and Section 3 first reports the unidimensional distributions of these direct coding dimensions. Several marginal results directly related to evidence continuity also show that real-world linkages are widely visible, while breakpoint states—including unidentifiable claim conditions, relevant conditions not entering testing, and non-codable uncertainty responses—also occur. Section 3.2 therefore summarizes this discontinuity in public evidential relationships as the physical fidelity gap (PFG) and confirms its existence but does not estimate the proportion of studies involving PFG or its degree. Section 4 then combines the relevant fields across dimensions for all 556 studies and operationalizes three evidence-traceability tiers—Strong, Medium, and Weak—that inversely correspond to PFG strength; Section 5 compares study characteristics not directly involved in the stratification and different outcome manifestations.

## 2. Materials and Methods

### 2.1. Literature Retrieval, Screening, and Corpus Construction

**Figure 1:**
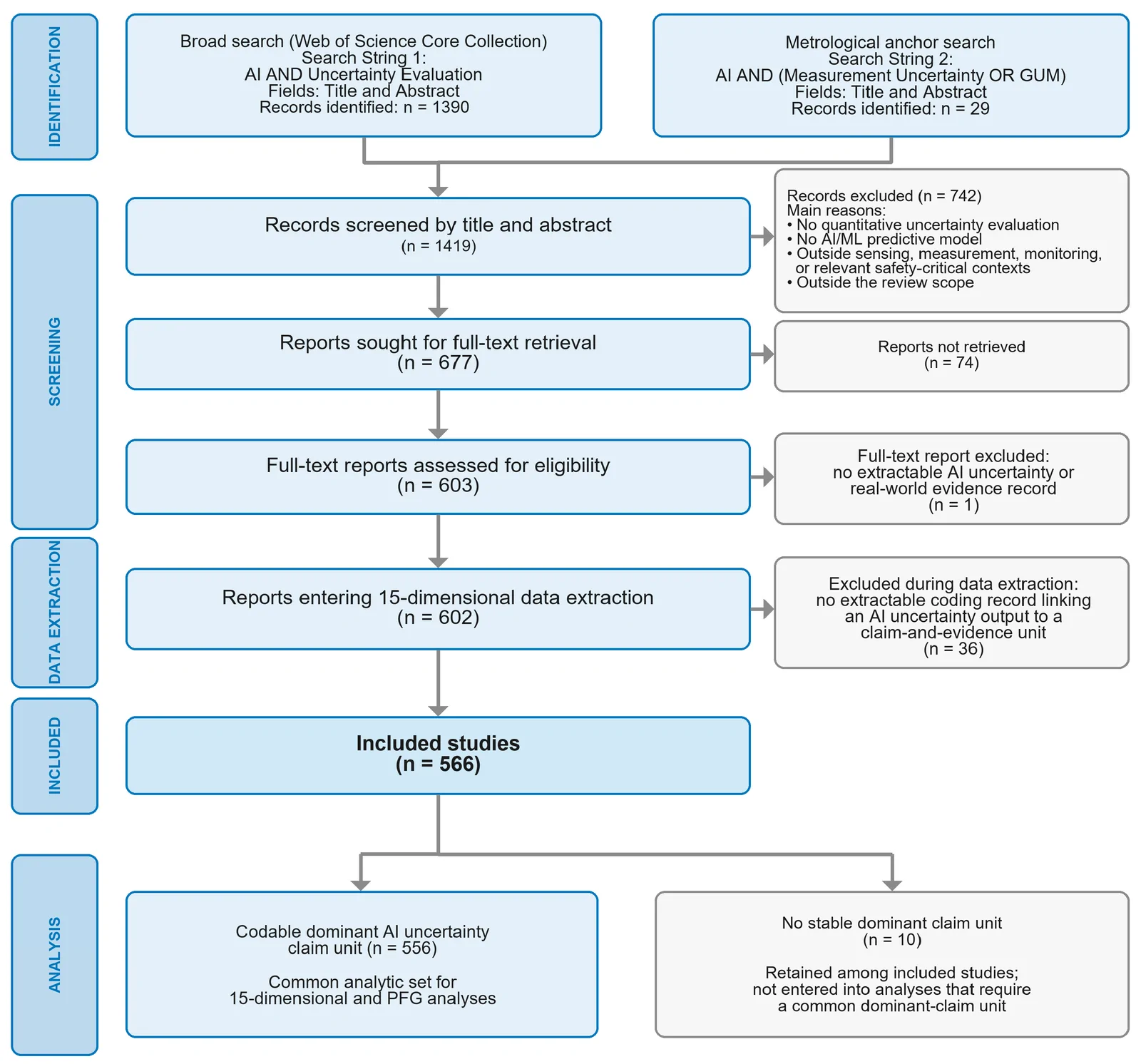
Study identification, screening, data extraction, and analysis workflow. The identification and screening stages were visualized using an adaptation of the general PRISMA 2020 flow structure [36]. The use of this structure is limited to workflow reporting and does not imply full PRISMA compliance. The lower stages are study-specific extensions used to distinguish the included studies (n = 566) from the common analytic set (n = 556).

The literature search used the Web of Science Core Collection as the data source and employed two complementary Boolean search strings, with Title and Abstract as the search fields for both. The first, broad search string was “AI AND Uncertainty Evaluation” and was used to cover studies on AI uncertainty estimation, calibration, reliability evaluation, and out-of-distribution evaluation related to sensing, measurement, monitoring, and related safety-critical settings. The second, metrology-anchored search string was “AI AND (Measurement Uncertainty OR GUM)” and was used to supplement studies explicitly involving measurement uncertainty, GUM-related terms, metrological validation, or standardized uncertainty evaluation. The broad search retrieved 1390 records, and the metrology-anchored search retrieved 29 records; a total of 1419 records entered title and abstract screening. PRISMA 2020 is a reporting guideline commonly used in systematic reviews and meta-analyses to make transparent how a broad literature set is narrowed through identification, screening, eligibility assessment, and final inclusion [36]. We therefore drew on this staged flow logic to document and visualize the literature identification and screening process. Because the present study is a structured coding review and does not implement every PRISMA requirement, this use is an adaptation and should not be interpreted as a claim of full PRISMA compliance. Figure 1 extends this adapted workflow beyond literature screening to show full-text data extraction and the formation of the analytic set.

Title and abstract screening excluded 742 records, mainly because they did not conduct quantitative uncertainty evaluation, did not use an AI/ML predictive model, involved a research setting outside sensing, measurement, monitoring, or a related safety-critical scope, or did not meet the scope of this review. The remaining 677 reports entered the full-text retrieval stage, and the full text could not be obtained for 74 of them. A total of 603 full-text reports were assessed for eligibility; 1 was excluded because it did not provide AI uncertainty or real-world evidence content from which a record could be formed within the data-extraction scope of this study, and the remaining 602 entered 15-dimensional full-text data extraction. During the 15-dimensional full-text data extraction, 36 studies failed to produce usable coding records: the articles did not establish a correspondence sufficient to generate a record between an AI uncertainty output and an identifiable uncertainty claim and its evidence items. Because these full texts did not yield the data records required for the subsequent synthesis, they were excluded for this common reason, leaving 566 included studies. Among the 566 included studies, 556 allowed one dominant AI uncertainty claim unit to be stably identified and coded and constituted the common analytic set for the 15-dimensional distributions, subsequent cross-dimensional synthesis, and category comparisons. The remaining 10 met the topical and full-text eligibility requirements but did not allow one dominant claim unit to be determined stably; their study IDs, reportable study characteristics, and missing or non-codable states were therefore retained, but they were not included in statistical analyses requiring a uniform dominant claim unit. This treatment distinguishes the 36 full texts that did not produce any record during data extraction from the 10 included studies that did not satisfy the requirements for a specific analytic unit.

### 2.2. Fifteen-Dimensional Data Extraction and Row-Level Evidence Traceability

Each included study was assigned a unique internal identifier (P0001–P0566) and treated as the basic unit of observation. For studies from which an analytic unit could be formed, one dominant AI uncertainty claim unit was identified in the full text; this unit is the set of text and results formed around the same AI uncertainty output that defines the claim object, applicable conditions, and primary evidence. When a study contained multiple uncertainty claims, the claim most directly related to the primary research objective and core empirical results was selected; the other claims were not repeatedly counted as independent observations, thereby maintaining “one primary analytic record per study.” The 10 studies for which a dominant claim could not be identified stably retained their internal identifiers and audit records but did not enter the common analytic set of 556 studies.

The 15 data-extraction dimensions were determined before category frequencies were summarized and cross-dimensional combinations were formed. They were designed to cover the basic information necessary for comparing AI uncertainty studies. Data extraction sequentially answered five basic questions: What uncertainty output did the study produce? What claim did the authors make about that output? To what real-world information and conditions of validity was the claim linked? Did those conditions enter empirical testing? What identifiable manifestation did the uncertainty output show during testing? To distinguish alternative explanations that might arise from model-performance evidence, reporting terminology, and implementation form, the framework further recorded the evidence target and validation protocol; the location at which uncertainty was formed and the related assumptions; the implementation path and computability motivation; and the claim use context and measurement interpretation level. These steps produced 15 fixed dimensions; the specific enumerated values converted these observational questions into consistent rules for full-text recording.

Table 1 presents the operational meaning of each data-extraction dimension and its analytical role in the subsequent synthesis. The table explains the correspondence between the observational fields and the full-text recording questions. The complete enumerated categories for each dimension are provided in the “Coding Dictionary” worksheet, the final code values for the 566 studies in the “Study Coding Matrix” worksheet, and the row-level evidence records supporting each code in the “Coding Evidence Chain” worksheet of Supplementary File S1.

All coding was based on statements, methods, experimental settings, tables, figures, or results that could be located in the full text. Each final code value corresponded to at least one row-level audit record. When a paper provided locatable text, the evidence form was recorded as “Verbatim Quotation”; when the final value was a missing information category such as Not reported, Not specified, or Not codable, a “Non-Reporting Note” was used to record the target content searched and the textual scope. The final code values are retained in the “Study Coding Matrix” worksheet and their supporting records in the “Coding Evidence Chain” worksheet of Supplementary File S1.

Data extraction and coding were implemented as a constrained, AI-assisted workflow. In July 2026, the retrieved PDFs were converted into searchable Markdown representations using Google Gemini 2.5 Pro through Vertex AI batch prediction (maximum output-token setting: 65,535). OpenAI Codex (a GPT-5-based agent; used in July-August 2026) was then used to locate candidate passages, organize them under the 15 predefined dimensions, and propose provisional values drawn only from the controlled coding dictionary. Each candidate record retained the Study ID, coding dimension and field, provisional code value, evidence form, verbatim quotation or non-reporting note, page or section, Evidence ID, and source file. The authors defined the coding dictionary and dominant claim rules, retained responsibility for resolving flagged or ambiguous cases, and approved the final study-level codes.

**Figure 2:**
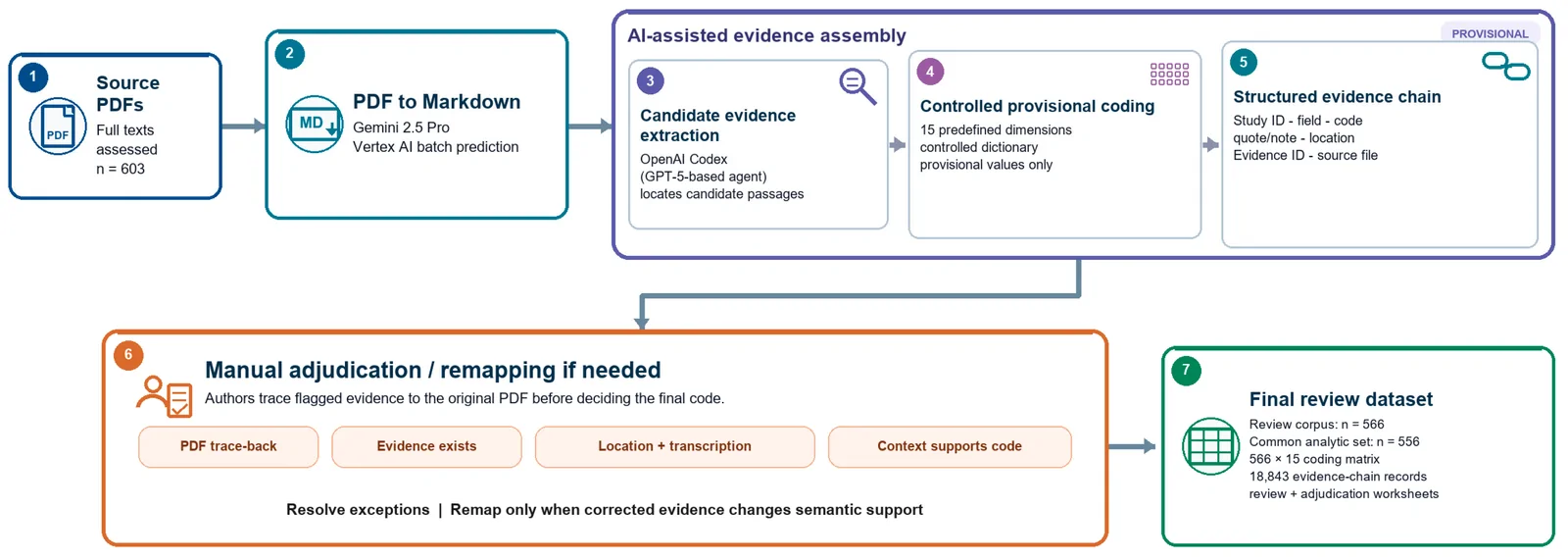
Operational workflow for AI-assisted full-text conversion, controlled evidence coding, structured evidence chain construction, and author adjudication. AI outputs remained provisional; the authors retained responsibility for final decisions and for any necessary remapping under the predefined enumeration strategy.

As summarized in Figure 2, manual adjudication included tracing each flagged evidence item back to the corresponding pages of the original PDF. Evidence verification then assessed four properties: whether the cited content existed in the source document, whether the recorded location was correct, whether the transcription was faithful, and whether the surrounding context supported the proposed code. AI assistance was used to organize and compare the evidence records, but the authors made the adjudication decisions. Records without an identified exception were retained through a rule-based pass procedure; records marked as corrected, escalated, or not found were handled separately. Corrections limited to page numbers, quotation form, or transcription did not automatically change the code. When a correction changed the semantic support, all valid evidence for the same study dimension was remapped under the original controlled enumeration strategy; insufficient or conflicting support was assigned the corresponding conservative non-identifiable or not-reported value. The resulting evidence-level review and study dimension adjudications are reported in the Manual Verification and Coding Adjudication Summary worksheets of Supplementary File S1. Because this was a traceability-based audit rather than an independent dual-coder design, no inter-rater agreement coefficient was calculated.

The study-level traceability tiers were assigned only after the direct fields had been coded; the sequential Q1–Q3 decision rules and corpus-based examples of complete and broken chains are presented in Section 4.

### 2.3. Three-Stage Evidence Synthesis and Analytical Strategy

This study used a sequential three-stage evidence synthesis. The first stage corresponds to Section 3. Using the 556 studies in the common analytic set as the denominator, the analysis reports the frequencies, proportions, and necessary category aggregates for 10 direct fields selected for stand-alone marginal profiling of uncertainty formation and observable claim–condition–test–response nodes. This selection was based on the analytic role rather than data availability; Section 3.1 explains how the other five dimensions are used in tier construction or tier-stratified analysis. Section 3 then uses several unidimensional marginal distributions directly related to the PFG definition to examine whether real-world linkages are present and whether unidentifiable, untested, or non-codable-response states occur at key evidential nodes. Section 3.2 uses these observations only to establish the existence of PFG in the current corpus; because the marginal distributions do not show whether these states overlap within the same study, that section does not estimate the proportion of studies involving PFG, judge its degree, or form tiers.

The second stage corresponds to Section 4. Based on the PFG definition formed in Section 3.2, direct coding fields are combined for the first time at the individual study level to establish three sequential evidence questions and thereby form the Strong, Medium, and Weak tiers; the specific field mappings and decision rules are given in Section 4 together with that analytical step.

The third stage corresponds to Section 5. This section uses the tiers formed in Section 4 as descriptive groupings, compares the nine coding dimensions not directly involved in stratification, and reports Robust/trigger-responsive uncertainty, Multiple uncertainty manifestations, and specific failure manifestations within Uncertainty_Manifestation as separate outcome directions. Interpretations directly corresponding to specific statistical patterns are also presented with the relevant analyses to form bounded mechanistic hypotheses and implications for improvement. The category comparisons present the combined Medium and Strong proportion, the Strong proportion, and the descriptive baseline for all 556 studies. The main text gives priority to categories with sample sizes of at least 15; small-sample categories and cross-dimensional configurations are treated only as exploratory indications.

## 3. Fifteen-Dimensional Coding Analysis of AI Uncertainty

### 3.1. Distributions of the Direct Coding Dimensions

Among the 566 included studies, 556 allowed the dominant uncertainty claim unit to be coded, accounting for 98.2% of all studies. This section uses these 556 studies as the statistical base and reports direct coding results that can be interpreted without cross-field derivation. Table 2 and Table 3 intentionally summarize the 10 dimensions selected for stand-alone marginal profiling of uncertainty formation and the observable evidence-chain nodes used to establish PFG, rather than reproducing all 15 fields in the main-text tables. The selection criterion was an analytic role, not data availability. These dimensions are grouped into two sets: the first includes uncertainty output type, claim object, entry point in the AI workflow, and related assumptions; the second includes claim-related physical information and conditions, condition testing, validation protocol, trigger type, and uncertainty manifestation. The other five dimensions were not omitted from analysis. Evidence_Target is one of the two direct inputs to Q2 in Section 4, together with Uncertainty_Manifestation; because it contributes to constructing the evidence-traceability tiers, comparing it again with the resulting tiers would be partly circular. Claim_Use_Context, Implementation_Channel_Change, Computability_Motivation, and Measurement_Interpretation_Level are contextual, engineering, or interpretive modifiers. Their main statistical use is the category-specific comparison of Medium + Strong and Strong proportions in Section 5, rather than an additional corpus-wide marginal table. Complete study-level values for all 15 dimensions are provided in the “Study Coding Matrix” worksheet of Supplementary File S1. The definitions and coding rules for each dimension are provided in Section 2.2 and in the “Coding Dictionary” worksheet of Supplementary File S1.

#### 3.1.1. Uncertainty Outputs, Claim Objects, and Formation Mechanisms

Uncertainty output types show a relatively dispersed distribution (Table 2). Prediction interval/set/bounds, the most frequent category, accounts for only 24.6% of all studies, indicating that current research has not formed one common dominant way of expressing uncertainty. The coexistence of intervals, variances, probabilities, entropy, predictive distributions, and various scores reflects differences in technical routes and also means that outputs termed “uncertainty” in different studies do not necessarily have the same statistical meaning. For example, explicit modeling of non-Gaussian aleatoric uncertainty shows that the statistical interpretation of an output distribution depends on the distributional structure used [37]. Only 13 studies, or 2.3%, directly use Measurement result uncertainty as the output type. This proportion indicates that few studies explicitly use measurement-uncertainty terminology to define model outputs, but it cannot be used to determine whether other outputs have measurement meaning; the latter also depends on the object, conditions, and validation evidence to which the output corresponds. Prediction reliability/confidence (34.2%), Model/epistemic uncertainty (18.3%), and Calibration/coverage reliability (16.5%) were the three most frequently coded claim objects and together accounted for 69.1% of the studies. By comparison, studies whose primary objects were Measurement result uncertainty, Deployment/decision risk, or Physical/parameter uncertainty together accounted for 9.5%. These results indicate that prediction reliability, model uncertainty, and calibration were the most frequently studied claim objects, while measurement, deployment/decision, and physical/parameter uncertainty were less common.

Multiple uncertainty entry points occur simultaneously in 61.7% of the studies. Most uncertainty outputs are not produced directly by one independent module and may instead be jointly affected by predictive distributions, posterior approximations, sampling or ensembles, proxy scores, and post-hoc calibration. This composite formation mechanism limits the interpretability of comparing studies only by the name of their final output; even if two studies both report predictive variance or confidence, their values may arise from different model mechanisms and have different applicable conditions. For example, the parallel evaluation of multiple post-hoc UQ approaches in SAR flood detection provides a specific case for distinguishing final outputs from generation mechanisms [38].

The identifiability of the related assumptions is uneven. In 32.0% of the studies, assumptions could not be assigned to an identifiable assumption category; among studies in which an assumption could be identified separately, Explicit likelihood/prior accounts for 27.2%, whereas Explicit sensor/physical model accounts for 6.5%. Here, “Not codable” means that the relevant assumptions were not reported with sufficient explicitness or could not be stably identified from the dominant claim unit. Overall, statistical assumptions internal to probabilistic models are more readily stated explicitly in papers than sensor mechanisms, physical processes, and data-generation mechanisms. Model discrepancy can also be treated as an independent source of uncertainty and propagated to downstream material properties [39].

#### 3.1.2. Physical Conditions, Validation Methods, and Uncertainty Manifestations

In 88.3% of the studies, at least one type of claim-related physical information or data-generation information could be identified. Physical information is therefore not generally absent from current AI uncertainty research, as shown in Table 3. For example, studies of seismic structures and source localization can incorporate physical information into uncertainty propagation [40], but this incorporation in itself still cannot replace direct testing of the claim-related conditions. In 72.1% of the studies, at least one physical, operational, or data-generation condition related to the uncertainty claim could be identified. This indicates that most studies do not discuss uncertainty entirely without conditional restrictions. Direct testing of claim-related conditions accounts for 41.7% of the studies, and indirect testing accounts for 46.0%. The combined high proportion of the two types of testing indicates that empirical evaluation is generally present in the literature; however, direct testing still does not constitute a majority of all studies.

Multiple validation protocols are used in 60.1% of the studies. A larger number of protocols indicates that studies usually do not rely on a single evaluation procedure, but it does not establish that the scope of validation covers actual measurement or deployment conditions. In 31.8% of the studies, no identifiable trigger test was reported or implemented. Among studies with one explicit trigger category, Unknown category/unseen class/OOD and Noise enhancement/SNR drop together account for 33.5%, whereas more specific physical triggers—including physical parameter changes, transient events, spatial extrapolation, cross-device tests, and cross-time drift—together account for 4.0%. Because some specific triggers may also be included in the Multiple physical triggers category, the latter proportion cannot be interpreted as their overall occurrence. Cross-dataset evaluation in remote photoplethysmography [41] and real-world data drift in welding quality monitoring [42] show that such triggers can be operationalized in specific applications.

The specific manifestation of uncertainty under trigger conditions is the least fully reported part of the above validation chain. In 45.1% of the studies, no codable uncertainty manifestation could be identified. This means that some studies tested the model or compared conditions but did not explicitly state whether the uncertainty output itself changed as expected when risk increased, the distribution changed, or physical conditions changed. Specific failure manifestations—including Calibration degradation, Overconfidence under shift, Under-coverage, OOD detection failure, Variance non-response, and Interval non-expansion/collapse—together account for 9.4% when they appear as a single primary category. An unreported uncertainty manifestation cannot be treated as an absence of failure, but it limits the reader’s ability to determine whether the model responded appropriately to the relevant conditions. Calibration under distribution shift has been specifically evaluated in fault diagnosis [43], and studies of efficient ensembles for industrial image classification have also directly compared uncertainty manifestations under in-distribution and OOD conditions [44].

Taken together, these direct coding results show that the existing literature has widely adopted uncertainty outputs and usually includes physical information, condition descriptions, and empirical validation. The marginal distributions also show that prediction, model, and calibration issues were the most frequently coded claim objects; although physical conditions occur frequently, direct condition testing does not yet constitute a majority; compared with general model evaluation, how uncertainty changes under trigger conditions is less often reported explicitly.

### 3.2. Existence of the Physical Fidelity Gap: Evidence from Unidimensional Coding

The physical fidelity gap (PFG) refers to the extent to which a continuous, verifiable evidential correspondence between an AI uncertainty output and the relevant real-world physical process is missing, untraceable, or incomplete. Within the analytical boundary of this study, PFG concerns only whether this correspondence can be traced continuously through evidence publicly reported in papers and does not infer unreported experimental or model facts. The unidimensional marginal distributions in Section 3.1 show, on the one hand, that real-world information is widely visible and, on the other hand, that states such as unidentifiable claim-related conditions, relevant conditions not entering testing, and non-codable uncertainty manifestations all occur with nonzero frequencies. These states, summarized in Table 4, are located respectively at the claim–condition, condition–test, and test–uncertainty-response nodes and are sufficient to show that the evidence breakpoint phenomenon constituting PFG does occur in the current corpus. Existence here is a corpus-level binary judgment: it does not determine which individual studies have a PFG, estimate the proportion of studies involving PFG, or judge its degree.

These unidimensional results are sufficient only to support that observable instances of the evidence breakpoint phenomenon indicated by PFG occur in the current corpus, and therefore that PFG is not a purely theoretical proposition; they are insufficient to determine how many studies have a PFG or what degree PFG reaches within an individual study. No single count is the number of PFG studies, and the proportions must not be combined. Section 4 therefore combines the relevant fields for the first time at the individual study level and converts the phenomenon-level existence observation into a verifiable evidence chain analysis and relative stratification; Section 5 then compares study patterns and outcome manifestations across tiers.

## 4. Evidence-Traceability Stratification of the Physical Fidelity Gap

The unidimensional marginal distributions in Section 3.2 established the existence of PFG but could not show whether the breakpoints co-occurred within the same study. This section therefore uses the 556 codable studies as a common analytic set, combines the relevant fields at the individual study level, and classifies the degree of PFG across studies. Strong, Medium, and Weak denote tiers of evidence traceability between AI uncertainty outputs and the relevant physical processes and thereby inversely characterize PFG strength: Strong indicates a relatively complete evidence chain and therefore corresponds to a weaker PFG; Medium indicates that the core evidence chain has been formed, but extended corroboration is limited and corresponds to a moderate PFG; Weak indicates that the evidence chain breaks earlier at condition testing or the uncertainty response links and corresponds to a stronger PFG.

This section selects six fields directly related to the evidence chain from the 15-dimensional coding and forms three sequential questions. Q1 combines Physical_Condition_For_Claim and Physical_Condition_Tested to determine whether the condition was identified and entered testing. Q2 combines Evidence_Target and Uncertainty_Manifestation to determine whether uncertainty-specific evidence and a codable manifestation are both present within the same dominant claim unit. Q3 uses Physical_Trigger_Type and Validation_Protocol to determine whether the existing evidence includes additional trigger breadth or extended validation. The specific rules are shown in Table 5.

These sequential, non-compensatory rules can be illustrated by two studies already included in the corpus. Pyle et al. [16] evaluated ultrasonic crack characterization using simulated and experimental crack data. An identifiable inspection condition entered testing, so Q1 was satisfied. However, within the coded dominant claim unit, calibration between uncertainty and crack-size prediction error was specified as an evaluation aim without a codable observed uncertainty response under the tested condition. The study therefore failed Q2 and was assigned to Weak.

In contrast, Jiang et al. [20] used QuaDRiGa-simulated channel data, trained on UMi line-of-sight and non-line-of-sight channel models, and tested on an out-of-distribution UMa non-line-of-sight model, thereby satisfying Q1. The study also reported a named uncertainty statistic, tau, and evaluated its thresholded association with qualified and unqualified channel state information predictions, satisfying Q2. Additional tests across antenna counts and user equipment speeds provided multiple trigger conditions, satisfying Q3 and assigning the study to Strong. This example also shows that real sensor data were not mandatory; simulated data were eligible when the simulated condition represented the claim-relevant physical or operational condition and the uncertainty response itself was evaluated. Thus, complete and broken refer to the continuity of the condition-test-uncertainty-response chain, rather than to whether an article or quotation could be traced to its source.

Among the 556 studies, 377 (67.8%) passed Q1, meaning that an identifiable relevant condition had entered testing directly or indirectly; 179 (32.2%) did not form this correspondence and entered Weak. Among the 377 studies that passed Q1, 209 (55.4%) also passed Q2 and formed identifiable evidence of an uncertainty response; the remaining 168 (44.6%) had condition testing, but the evidence target or uncertainty manifestation was insufficient to determine how uncertainty responded, and therefore also entered Weak. Among the 209 studies that passed the first two questions, 76 (36.4%) also had additional trigger breadth or extended validation corroboration and were assigned to Strong; the remaining 133 (63.6%) were assigned to Medium. Finally, 347 studies were classified as Weak, accounting for 62.4%; 133 were classified as Medium, accounting for 23.9%; and 76 were classified as Strong, accounting for 13.7%. The final results are shown in Table 6.

This section operationalizes PFG as an evidence-traceability gap and accordingly provides a relative stratification by strength. The stratification uses quantitative counts to present relative tier differences in the completeness of the evidence chain.

## 5. Research Patterns and Outcome Manifestations Across PFG Evidence-Traceability Tiers

### 5.1. Analysis Strategy and Comparison Benchmarks

**Figure 3:**
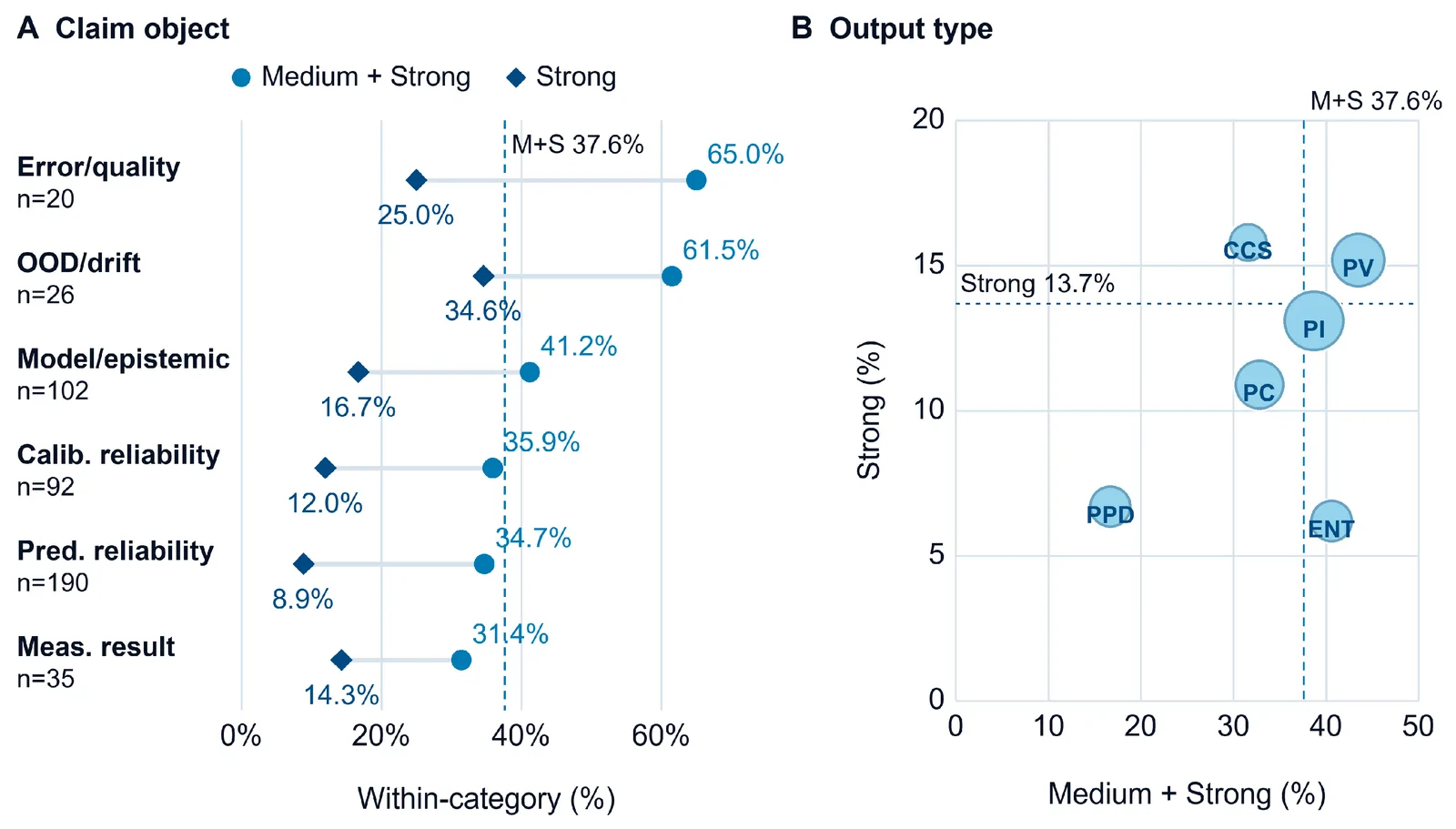
Distribution of PFG evidence-traceability tiers across Uncertainty Claim Object and Uncertainty Output Type. (**A**) Uncertainty Claim Object; (**B**) Uncertainty Output Type. Each proportion uses n for the corresponding category as its denominator. Labels in panel (**A**): Error/quality = Error/quality estimation; OOD/drift = OOD/drift risk; Model/epistemic = Model/epistemic uncertainty; Calib. reliability = Calibration/coverage reliability; Pred. reliability = Prediction reliability/confidence; Meas. result = Measurement result uncertainty. Codes in panel (**B**): PV = Predictive variance/dispersion; PI = Prediction interval/set/bounds; PC = Predictive confidence/probability; ENT = Entropy/information measure; PPD = Posterior/predictive distribution; CCS = Calibration/coverage score. Across all panels, circles show Medium + Strong, diamonds show Strong, vertical dashed lines show the corresponding overall benchmarks, and n is the category denominator. The overall benchmarks for Medium + Strong and Strong were 37.6% and 13.7%, respectively. Complete values are provided in the ‘Study Coding Matrix’ worksheet of Supplementary File S1.

**Figure 4:**
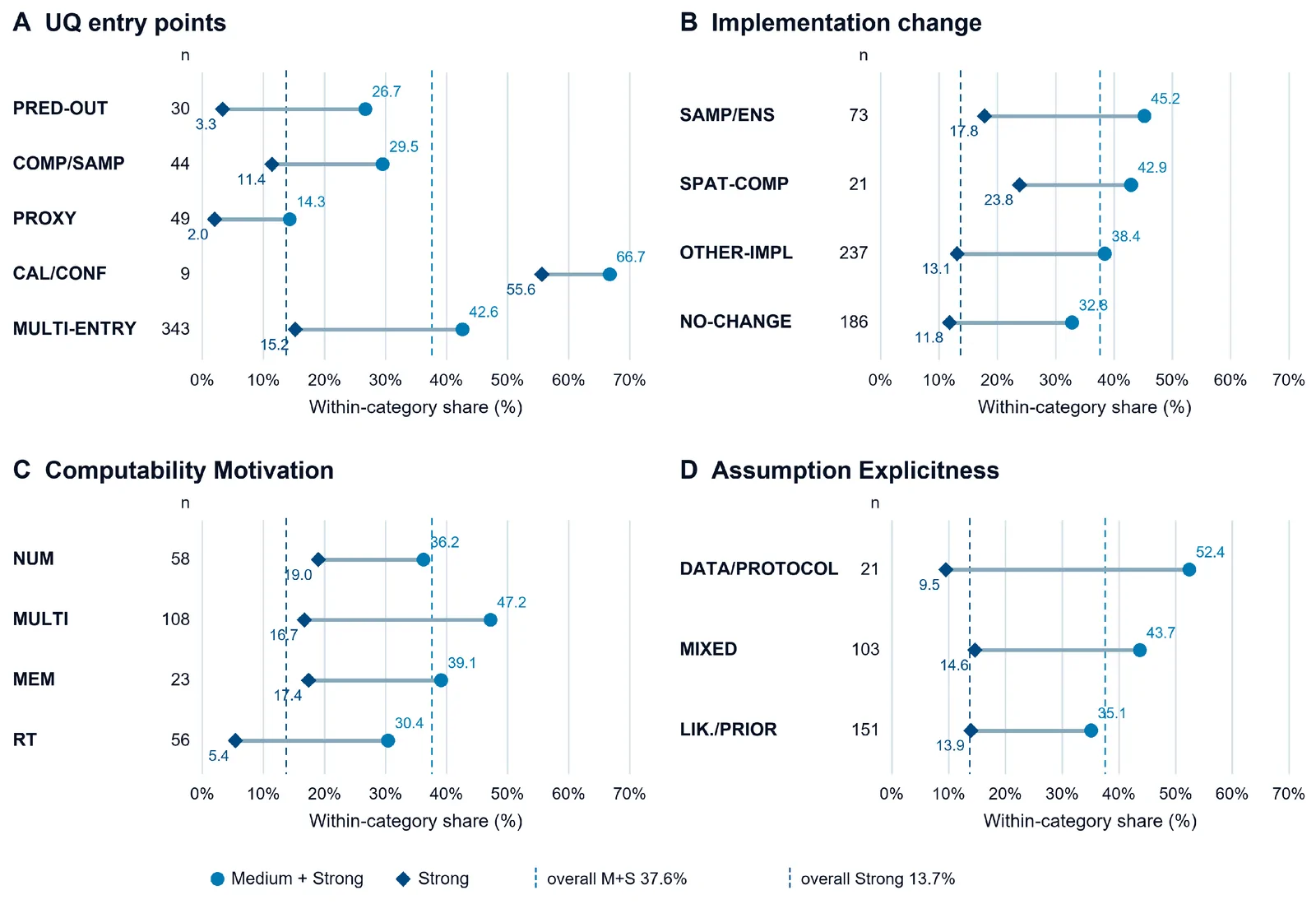
Distribution of PFG evidence-traceability tiers across AI Uncertainty Entry Point, Implementation Channel Change, Computability Motivation, and Assumption Explicitness. (**A**) AI Uncertainty Entry Point; (**B**) Implementation Channel Change; (**C**) Computability Motivation; (**D**) Assumption Explicitness. Codes in panel (**A**): PRED-OUT = Predictive likelihood/output distribution; COMP/SAMP = Computational approximation/sampling; PROXY = Uncertainty proxy/score; CAL/CONF = Calibration/conformal interface; MULTI-ENTRY = Multiple uncertainty entry points. Codes in panel (**B**): SAMP/ENS = Sampling/ensemble approximation; SPAT-COMP = Spatial/local-context compression; OTHER-IMPL = Multiple/other implementation changes; NO-CHANGE = No separate implementation change/not applicable. Codes in panel (**C**): NUM = Numerical tractability/stability; MULTI = Multiple computability motivations; MEM = Memory/dimensionality; RT = Latency/real-time inference. Across all panels, circles show Medium + Strong, diamonds show Strong, vertical dashed lines show the corresponding overall benchmarks, and n is the category denominator. Labels in panel (**D**): Data/protocol = Explicit data/protocol assumption; Mixed = Mixed/multiple assumptions; Likelihood/prior = Explicit likelihood/prior. The CAL/CONF category included only 9 studies, and the corresponding proportions are presented only as exploratory indications. Complete values are provided in the ‘Study Coding Matrix’ worksheet of Supplementary File S1.

**Figure 5:**
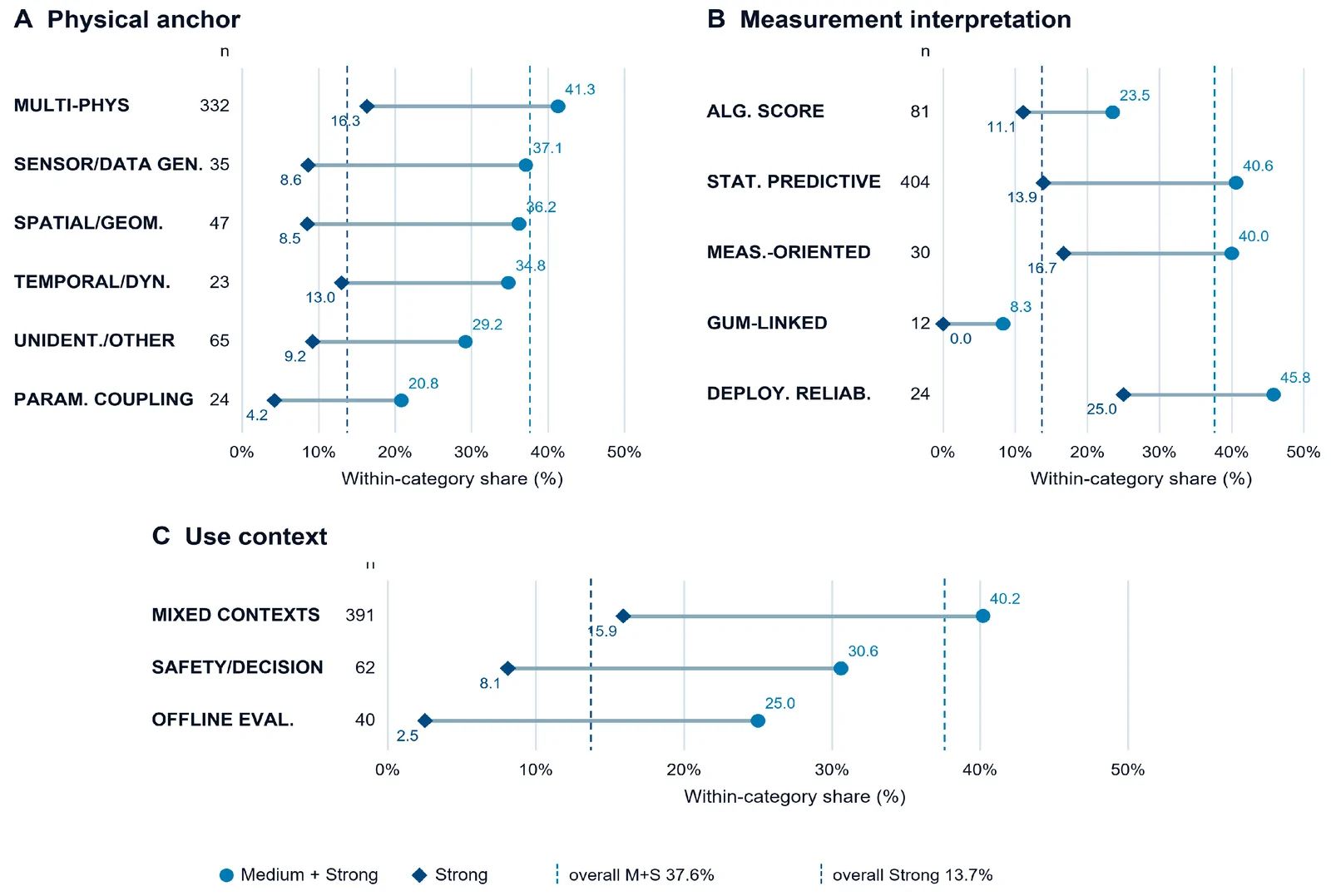
Distribution of PFG evidence-traceability tiers across Physical Information Linked to the Claim, Measurement Interpretation Level, and Claim Use Context. (**A**) Physical Information Linked to the Claim; (**B**) Measurement Interpretation Level; (**C**) Claim Use Context. Labels in panel (**A**): MULTI-PHYS = Multiple physical information types; SENSOR/DATA GEN. = Sensor/data-generation mechanism; SPATIAL/GEOM. = Spatial/geometric structure; TEMPORAL/DYN. = Temporal/dynamic structure; UNIDENT./OTHER = Not identifiable/other; PARAM. Coupling = Parameter dependency/coupling. Labels in panel (**B**): ALG. SCORE = Algorithmic uncertainty score; STAT. PREDICTIVE = Statistical predictive uncertainty; MEAS.-ORIENTED = Measurement-oriented uncertainty claim; GUM-LINKED = Formal measurement uncertainty/GUM-linked; DEPLOY. RELIAB. = Physical deployment reliability. Labels in panel (**C**): MIXED CONTEXTS = Multiple/mixed use contexts; SAFETY/DECISION = Safety/decision support; OFFLINE EVAL. = Offline evaluation/comparison. The GUM-linked category included only 12 studies and should be interpreted cautiously in light of the small sample. Complete values are provided in the ‘Study Coding Matrix’ worksheet of Supplementary File S1.

**Figure 6:**
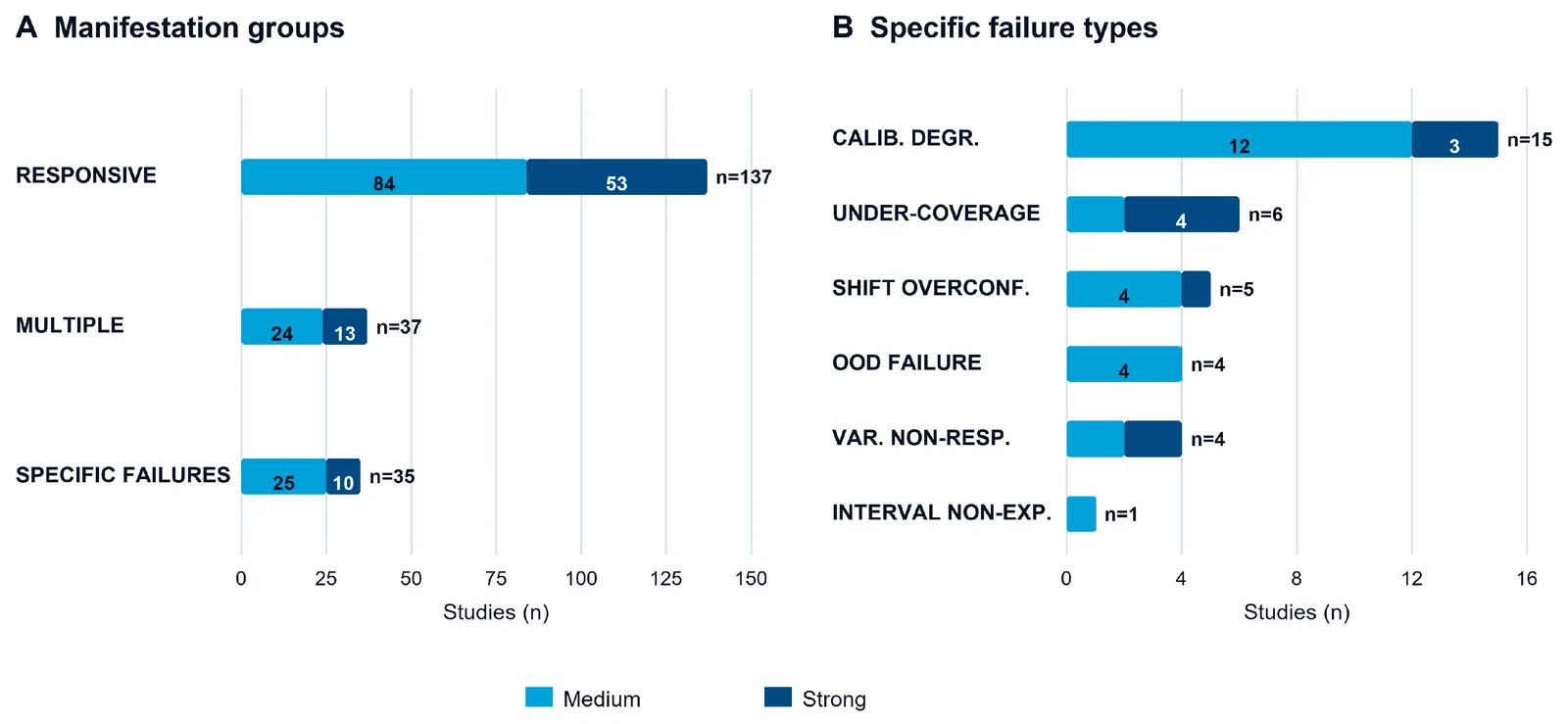
Uncertainty manifestation in Medium and Strong studies. (**A**) Medium/Strong counts across three manifestation groups; (**B**) Medium/Strong counts across six specific failure types. Both panels use the same stacked-bar grammar; horizontal scales differ because panel (**A**) reports aggregates and panel (**B**) reports the specific-failure breakdown. In panel (**A**), RESPONSIVE = Robust/trigger-responsive uncertainty, MULTIPLE = Multiple uncertainty manifestations, and “SPECIFIC FAILURE” denotes the sum of the six failure types shown in panel (**B**). Labels in panel (**B**): CALIB. DEGR. = Calibration degradation; UNDER-COVERAGE = Under-coverage; SHIFT OVERCONF. = Overconfidence under shift; OOD FAILURE = OOD detection failure; VAR. NON-RESP. = Variance non-response; INTERVAL NON-EXP. = Interval non-expansion/collapse. ‘Multiple uncertainty manifestations’ may include both robust and failure outcomes; an explicit failure is a negative physical-fidelity outcome and is not equivalent to PFG itself. Complete values are provided in the ‘Study Coding Matrix’ worksheet of Supplementary File S1.

**Figure 7:**
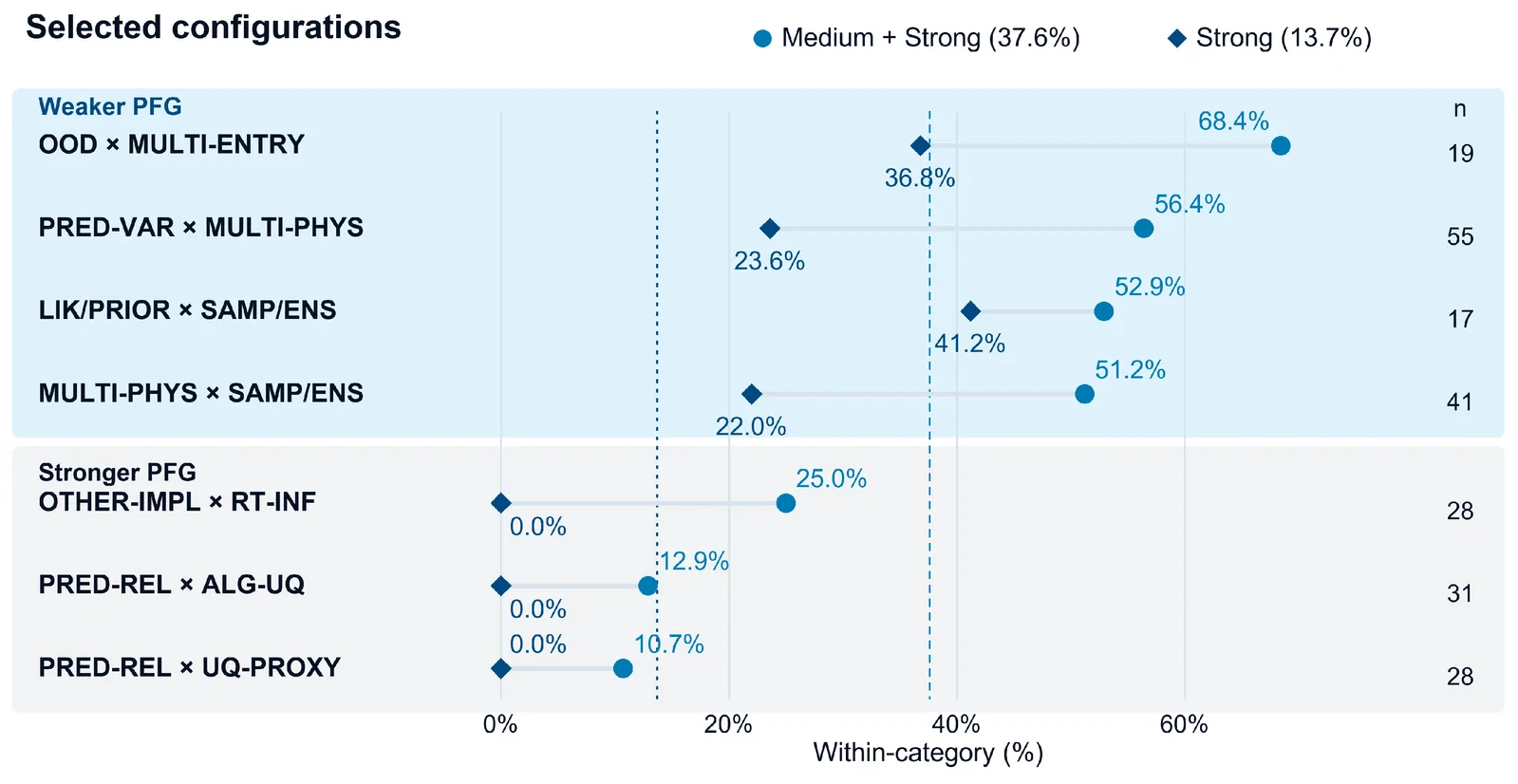
Distribution of PFG evidence-traceability tiers across selected cross-dimensional research configurations. The symbol × denotes a combination of categories within the same configuration, not mathematical multiplication. Codes: OOD = OOD/drift risk; MULTI-ENTRY = Multiple uncertainty entry points; PRED-VAR = Predictive variance/dispersion; MULTI-PHYS = Multiple physical information types; LIK/PRIOR = Explicit likelihood/prior; SAMP/ENS = Sampling/ensemble approximation; OTHER-IMPL = Multiple/other implementation changes; RT-INF = Latency/real-time inference; PRED-REL = Prediction reliability/confidence; ALG-UQ = Algorithmic uncertainty score; UQ-PROXY = Uncertainty proxy/score. The dashed lines indicate the overall benchmarks for Medium + Strong (37.6%) and Strong (13.7%), respectively. Weaker PFG and Stronger PFG indicate only the directions of these post hoc descriptive configurations relative to the overall benchmarks and are not used for causal inference or general classification. Complete values are provided in the ‘Study Coding Matrix’ worksheet of Supplementary File S1.

This section reports the proportions of Medium + Strong (hereafter M + S) and Strong within each category in Figure 3, Figure 4, Figure 5, Figure 6 and Figure 7. M + S denotes studies in which the core evidence chain is traceable, whereas Strong denotes the relatively most complete evidence chain under the current rules; the overall benchmarks were 37.6% (209/556) and 13.7% (76/556), respectively. All comparisons are unadjusted descriptive associations and are not used for causal judgment or method ranking.

To avoid circular interpretation, the six evidence dimensions used directly to define the tiers in Section 4 are not treated as the main comparison variables in this section; Uncertainty_Manifestation is used only in Section 5.5 to describe outcome direction. The remaining nine coding dimensions are used for the comparisons in Figure 3, Figure 4 and Figure 5 and Figure 7.

The main text focuses on categories with n ≥ 15, and small samples are used only for exploratory indications. The figures consistently report within-category proportions rather than the composition within the Strong tier to reduce misinterpretation caused by differences in category size.

This comparison strategy prioritizes identifying which observable research configurations co-occur with more complete evidence chains, without reinterpreting the fields used to define the tiers as causes of tier formation. Explicit category denominators, sample thresholds, and small-sample handling allow the basis of comparison to be checked and reduce the risk of overinterpreting extreme proportions. Future studies can use hierarchical models, covariate adjustment, or sensitivity analysis in an independent corpus to test whether these descriptive patterns remain stable.

### 5.2. Uncertainty Claim Object and Uncertainty Output Type

Figure 3 indicates that evidence traceability was associated more strongly with the specificity of the claim object than with the nominal form of the uncertainty output. Error/quality and OOD/drift claims reached M + S/Strong proportions of 65.0%/25.0% and 61.5%/34.6%, respectively, clearly above the overall benchmarks (37.6%/13.7%), whereas the broader Prediction reliability category was lower (34.7%/8.9%). This contrast suggests that a defined error or drift target makes it easier to align a test condition, an uncertainty response, and an evaluation endpoint. In contrast, output types did not show a stable ordering across M + S and Strong, and the Posterior/predictive distribution category was below both benchmarks (16.7%/6.7%). The pattern in Figure 3 shows that higher evidence traceability is associated with an explicit connection between the uncertainty output, a clearly specified risk object, and a testable evaluation criterion. For research design, variance, interval, probability, or entropy outputs should therefore be connected before modeling to the error, drift, or coverage deviation they are expected to detect, together with a verifiable response or failure threshold. A benchmark of deep-learning prognostics provides a concrete example; multiple UQ methods were compared under common tasks and perturbation conditions [45], allowing performance differences to be interpreted against the same risk and trigger structure.

### 5.3. AI Uncertainty Entry Point, Implementation Channel Change, and Computational Constraints

Figure 4 associates higher evidence traceability with research designs that expose multiple, testable interfaces. MULTI-ENTRY was above the overall M + S/Strong benchmarks (42.6%/15.2%), whereas PROXY was well below them (14.3%/2.0%); the high CAL/CONF estimate was based on only nine studies and remains exploratory. This contrast indicates that a stand-alone score provides limited evidential structure unless it is connected to a trigger and an evaluation endpoint. SAMP/ENS (45.2%/17.8%) and SPAT-COMP (42.9%/23.8%) were also above the benchmarks, whereas NO-CHANGE was lower (32.8%/11.8%). Sampling, ensembling, and local-context transformations therefore provide observable interfaces for ablation, coverage-deviation testing, or controlled perturbation, while their evidential contribution still depends on demonstrating an uncertainty-specific response. A heterogeneous ensemble for atomistic foundation models illustrates this mechanism by constructing a shared uncertainty metric and validating its correspondence with prediction error [46]. Real-time inference was lower (30.4%/5.4%), a pattern that may reflect limited validation depth, trigger scope, or reporting space under resource constraints. Distributed UQ [47] and submillisecond sensor demodulation at the edge [48] show how latency and computational capacity can be stated as explicit implementation conditions; reporting computational budget, task performance, and uncertainty response together makes their evidential role assessable. The divergent M + S and Strong positions of Data/protocol assumptions (52.4%/9.5%) further indicate that explicit assumptions improve core traceability more readily than extended corroboration.

### 5.4. Physical Information Linked to the Claim and Measurement Interpretation Level

Figure 5 shows that physical anchoring contributed most clearly when it was connected to deployment or to multiple mutually constraining information sources. Multiple physical information types were above the benchmarks (41.3%/16.3%), whereas Parameter dependency/coupling was the lowest category (20.8%/4.2%); this contrast suggests that combining sensor, spatial, temporal, or data-generation information creates more opportunities to test the same claim from complementary directions. Physical deployment reliability had the highest well-supported M + S/Strong proportions in the measurement-interpretation panel (45.8%/25.0%), while the Algorithmic uncertainty score was lower (23.5%/11.1%). The difference indicates that an uncertainty output becomes more traceable when the deployment condition, reference basis, and expected consequence are specified. The GUM-linked estimate (8.3%/0%) came from only 12 studies and is interpreted cautiously. In the use-context panel, Multiple/mixed contexts were above both benchmarks (40.2%/15.9%), whereas Safety/decision support (30.6%/8.1%) and Offline evaluation/comparison (25.0%/2.5%) were lower, showing that a declared application contributes limited evidence until its conditions and consequences enter testing. In-field calibration of low-cost particulate matter sensors [49] and the observation of residual random errors in smartphone infrared camera calibration [50] illustrate how reference measurements, error sources, and applicable conditions turn physical or metrological context into operational evidence. Across the three panels, the more complete chains connect a mechanism or condition to a test, an uncertainty response, and an observable consequence.

### 5.5. Physical Fidelity Outcomes Revealed by the Medium and Strong Evidence-Traceability Tiers

Figure 6 shows that more complete evidence chains primarily made outcome direction visible. Among the 209 Medium or Strong studies, 137 (65.6%) reported robust or trigger-responsive uncertainty, while 35 (16.7%) explicitly exposed failures; 10 of those failures occurred in the Strong tier. The presence of failures in Strong studies confirms that this tier captures the completeness and visibility of the evidence relationship; it does not certify positive method performance. Calibration degradation was the most frequent specific failure (15 studies), and Under-coverage appeared more often in Strong than Medium studies (4 versus 2), illustrating how extended testing can reveal limitations that remain hidden in less complete evaluations. These results support prespecifying negative endpoints such as calibration degradation, under-coverage, overconfidence under shift, and variance non-response, together with the trigger intensity, sample size, and detection sensitivity needed to expose them. A reported absence of failure is informative only when the test had sufficient power to detect the expected problem. The evidential value of an experiment therefore depends on whether positive and negative endpoints were defined and reported under conditions capable of discriminating the uncertainty response.

### 5.6. Cross-Dimensional Research Configurations

Figure 7 identifies combinations in which the relationship between risk object, uncertainty mechanism, and physical condition testing was especially clear. OOD × MULTI-ENTRY (68.4%/36.8%), PRED-VAR × MULTI-PHYS (56.4%/23.6%), and LIK/PRIOR × SAMP/ENS (52.9%/41.2%) were all well above the overall M + S/Strong benchmarks. Each configuration joins a specific claim with an identifiable uncertainty-generation mechanism or physical anchor, giving the trigger and response a common interpretation. In contrast, PRED-REL × ALG-UQ (12.9%/0%) and PRED-REL × UQ-PROXY (10.7%/0%) were far below the benchmarks; general reliability claims paired with independent scores or proxies provide fewer observable links unless the score is calibrated to an operational threshold, consequence, or target condition. The combined pattern supports a three-layer research design consisting of a specific risk object, an explicit uncertainty mechanism, and a physical condition test with defined endpoints. The configurations were selected post hoc from groups with at least 15 studies. They therefore provide hypotheses for preregistration or independent validation, and causal ranking of techniques is outside the scope of this analysis.

Across Figure 3, Figure 4, Figure 5, Figure 6 and Figure 7, higher evidence traceability co-occurred most consistently with designs that specified a concrete risk, exposed the uncertainty-generation mechanism, and tested its response under an explicit physical or operational condition. Their interpretation is limited to configurations for further validation; technology and application field rankings are outside the scope of this analysis.

## 6. Discussion and Conclusions

Section 3, Section 4 and Section 5 have already provided local discussions in conjunction with their direct results; this section provides only an integrated inference from the overall evidence chain and its formation mechanisms and, on this basis, summarizes the boundaries and contributions of this study.

### 6.1. Evidence Problems in Most Studies and Their Formation Mechanisms

The most direct integrated conclusion of this study is that most published studies have not yet closed, within the paper, the evidence chain required by their dominant claim. Among the 566 included studies, 556 could form dominant claim analysis units; according to the three sequential nodes of condition testing, uncertainty response, and extended corroboration, 347 studies were classified as Weak, accounting for 62.4%, 133 as Medium, accounting for 23.9%, and 76 as Strong, accounting for 13.7%. Because Weak means that the evidence chain breaks at least at Q1 or Q2, this result quantitatively indicates that the lack of a traceable correspondence between AI uncertainty outputs and real-world conditions is not an isolated occurrence in the present corpus, but a systematic phenomenon in the publicly reported evidence. Here, ‘problem’ strictly refers to the failure of the claim–evidence relationship presented in the paper to close and must not be extended to mean that the algorithm is wrong or the study has no value.

This conclusion is important because the literature does not generally lack physical information, condition descriptions, or empirical evaluations. Identifiable physical or data-generation information was present in 88.3% of studies, 72.1% allowed conditions related to the claim to be identified, direct and indirect condition testing accounted for 41.7% and 46.0%, respectively, and 60.1% also used multiple validation protocols. The actual break lies in whether these elements form a connection around the same uncertainty claim: 179 studies did not form the correspondence in which an identifiable claim-relevant condition entered testing; another 168 studies had claim-relevant conditions enter testing but did not form codable evidence of an uncertainty-specific response. Among all 377 studies that passed Q1, the latter category accounted for 44.6%. Therefore, the main gap in the current field is that the conditions changed in the experiment, the endpoints observed, and the real-world meaning carried by the uncertainty output are not explicitly aligned.

This break can be explained by the common organization of existing papers. Many studies first select variance, intervals, probabilities, entropy, or proxy scores and then use random splits, overall performance, or generic out-of-distribution tests to demonstrate model performance. When physical information is used only as an input, feature, constraint, or application context, it serves as a modeling resource rather than an evidential obligation; when uncertainty is used only for screening, weighting, ranking, or decision-making, while the results report only accuracy, error, or AUC, the study can demonstrate that the module ‘was used’ but does not necessarily demonstrate that the claim it supports ‘was tested.’ This explains why adding physical variables, validation repetitions, or model complexity does not automatically reduce PFG.

In data-driven AI, assumptions can enter diffusely through data, model, and implementation interfaces. Explicit assumptions can be written into the data and protocol layers, including the support range of training samples, data partitioning, labeling rules, and the construction of perturbations or OOD data; they can also be written into the model and objective layers, including the likelihood, prior, loss, and predictive distribution, and enter actual computation through posterior approximation, sampling, ensembling, local compression, or post hoc calibration. Implicit assumptions are often embedded in default correspondences, such as treating offline samples as representative of the deployment distribution, treating labels as a sufficient reference, or interpreting changes in variance, entropy, and proxy scores as changes in real-world risk. The phenomenon revealed by shortcut learning, in which a model is effective within a benchmark but fails outside its context, shows that stable correlations in training data do not necessarily constitute a stable basis in the target context [51]. In Section 5, the M + S/Strong proportions for Data/protocol, Mixed, and Likelihood/prior were 52.4%/9.5%, 43.7%/14.6%, and 35.1%/13.9%, respectively, and did not form a monotonic relationship from degree of formalization to evidence tier; different output types also showed no consistent advantage. These descriptive distributions are compatible with one interpretation: only when the correspondence among data range, model approximation, output semantics, and target conditions is converted into testable changes and the uncertainty output forms an identifiable response do the relevant premises move from modeling choices to traceable evidence.

This distributed mode of entry also explains the common direction across the dimensions in Section 5. The M + S/Strong proportions for Error/quality and OOD/drift were 65.0%/25.0% and 61.5%/34.6%, respectively, above the 34.7%/8.9% for Prediction reliability; MULTI-ENTRY was 42.6%/15.2%, whereas PROXY was 14.3%/2.0%; SAMP/ENS was +7.6/+4.1 percentage points relative to the overall M + S/Strong benchmarks, whereas real-time inference was 30.4%/5.4%. Real-world anchoring showed a similar direction: Multiple physical information types, Physical deployment reliability, and Multiple/mixed use contexts were 41.3%/16.3%, 45.8%/25.0%, and 40.2%/15.9%, respectively, whereas Algorithmic uncertainty score and Offline evaluation/comparison were 23.5%/11.1% and 25.0%/2.5%. Together, these directions suggest that specific risk objects, multiple entry points, and explicit target contexts more readily operationalize premises in data support, model choices, and implementation approximations into an observable ‘relevant condition–trigger–uncertainty response’ relationship; general reliability claims, a single proxy score, or overall offline performance more readily leave this correspondence at the level of a default assumption. Sampling, ensembling, and calibration provide implementation interfaces for establishing the correspondence, and existing research also shows that ensemble size and post hoc calibration change the reliability of uncertainty estimation [52]; however, the evidential meaning of an implementation choice still depends on whether the corresponding condition enters dedicated testing and whether the uncertainty output itself forms an identifiable response. Among the 209 Medium or Strong studies, 35 still explicitly exposed failures, indicating that a more complete evidence chain increases the visibility of assumptions and applicability boundaries but does not guarantee method success. Thus, the statistical results in Section 5 locate the ways in which assumptions enter AI uncertainty computation at interconnected interfaces, including data support, task and output definition, model and implementation approximation, and validation design, and show that these interfaces can be converted into more complete public evidence only when they form a verifiable correspondence around the same dominant claim.

### 6.2. What Kind of ‘Quality’ Does the Evidence Stratification Reflect?

Strong, Medium, and Weak do reveal clear tier differences in current research on AI uncertainty quantification, but the ‘quality’ that can be discussed here is limited to the completeness with which the dominant claim receives real-world evidence support and cannot substitute for the overall quality of the paper, algorithmic performance, or research value. Strong means that, under the rules of this study, conditions, testing, uncertainty responses, and additional corroboration form a relatively complete and readily verifiable chain; Medium means that the core chain has formed but extended corroboration is limited; Weak means that the chain breaks earlier. The three tiers correspond inversely to PFG strength but do not constitute a success–failure ranking. Among the 76 Strong studies, 10 still explicitly exposed a single uncertainty failure, which precisely indicates that stronger evidence traceability increases the visibility of failure rather than guaranteeing a positive result.

This stratification can be understood as normal heterogeneity while the field remains at a stage in which evidence norms are gradually taking shape, but it should not be stated as ‘there is no authoritative method in metrology.’ Metrology has already established a formal framework for measurement uncertainty through the GUM [34]; Carratù et al. further discussed the propagation of input measurement uncertainty in artificial neural networks [35]. What is still lacking is an authoritative evaluation specification that can uniformly connect claims, real-world conditions, and empirical responses across AI tasks, output types, and application contexts. Existing UQ reviews have systematically organized probabilistic, non-probabilistic, and propagation techniques [30] and have further summarized uncertainty-based multidisciplinary design optimization into surrogate modeling, decomposition, intelligent optimization, and other routes [31]. PFG stratification supplements the evidence question after the adoption of a technique: regardless of the method used to generate an output, can the real-world claim it supports be traced, tested, and verified?

Therefore, the contribution of this study is not to propose an audit standard for judging whether a study is ‘qualified or unqualified’ but to convert the previously general statement that ‘AI uncertainty evidence varies in completeness’ into observable, decomposable, and comparable reference coordinates. A study falling into Weak indicates that at least one key evidence node has not yet closed and usually warrants targeted addition of condition testing, uncertainty response, or extended corroboration; a study entering Strong indicates only that the organization of its public evidence is relatively complete and cannot further certify that the computation is correct, the statistical inference is valid, the setting is generalizable, or the paper is of high quality. PFG thus has the asymmetric property of being ‘sensitive to defects and incapable of certification’: it is more suitable for locating positions that need improvement.

### 6.3. Conclusions

This study applied a structured 15-dimensional coding framework to 566 studies on AI uncertainty quantification and used the 556 studies with identifiable dominant claims as the common analytic set. The results show that 347 studies (62.4%) experienced an evidence chain break either at the identification or testing of claim-relevant conditions or at the uncertainty response link; among them, 179 studies did not form a closed correspondence at the identification of claim-relevant conditions or the entry of the condition into testing, whereas 168 studies broke at the test–uncertainty response link. This study thereby quantitatively confirms that, in the present literature corpus, the non-closure of publicly reported evidence between AI uncertainty outputs and real-world physical, measurement, or operational conditions is not an isolated phenomenon, but a systematic issue that most studies need to address.

The resulting Weak, Medium, and Strong tiers reflect differences in the completeness of claim–evidence support in the current field. Given that AI uncertainty outputs still lack a unified authoritative evaluation specification across tasks and output types, this heterogeneity has a realistic basis; however, it does not mean that all tiers are equally sufficient, nor does it equate the PFG tier with the overall quality of a paper. Weak indicates that at least one key node in the evidence chain still needs to be strengthened, whereas Strong indicates only that the evidence relationship is more complete and more readily verifiable under the current reference frame.

The category-specific tier distributions further indicate how these differences may arise. Specific OOD, drift, error, or quality claims, as well as Multiple uncertainty entry points, Multiple physical information types, and Sampling/ensemble approximation, more often co-occurred with Strong; general prediction reliability, independent proxy scores, and real-time implementation constraints more often co-occurred with Weak. These patterns do not constitute rankings of technologies or fields but provide a clear direction for improvement: studies should not only increase output complexity or the number of validations but should move toward a closed chain of ‘specific claim–relevant condition testing–uncertainty-specific response–extended corroboration.’

The core contribution of this study is therefore to provide a bounded evidence reference frame: it converts evidence defects in AI uncertainty quantification that were previously difficult to locate into enumerable breaks, comparable levels, and discussable research configurations. This reference frame can indicate where a study is most likely to need strengthening but cannot be used to certify that a method is correct, a study is qualified, or a paper is of high quality. The value of PFG lies in making ‘whether this uncertainty claim has received real-world support’ an answerable question, rather than replacing independent evaluations of algorithmic performance, statistical validity, and metrological conformity.

This review did not conduct a separate venue-oriented search of conference proceedings. The corpus was intentionally limited to journal studies identified through the Web of Science Core Collection for which full texts could be retrieved for row-level evidence coding. This scope improved consistency in source retrieval and evidence traceability across the 15 coding dimensions, while the resulting 566-study corpus provided a substantial basis for the intended descriptive analysis. However, it may underrepresent recent methodological developments disseminated primarily through conference venues such as UAI, AISTATS, and AAAI. The findings should therefore be interpreted as characterizing the defined journal-literature corpus rather than the complete literature on AI uncertainty quantification.

## Figures and Tables

**Table 1:** Operational meanings of the 15 coding dimensions and their analytical roles in the review.

Coding Dimension	Operational Meaning and Rationale for Inclusion
Uncertainty_Output_Type	Records the mathematical or reported form of the AI uncertainty output, such as an interval, variance, distribution, probability, or score; used to separate the output form from the claim meaning assigned to that output by the authors.
Uncertainty_Claim_Object	Records the object that the authors claim the uncertainty output can characterize, evaluate, or assure; used to clarify whether the study discusses prediction reliability, model uncertainty, calibration coverage, measurement results, or deployment risk.
Claim_Use_Context	Records the context in which the uncertainty claim is used, such as prediction, calibration, offline comparison, safety decision-making, or deployment; used to define the use and applicable consequences of the claim.
Evidence_Target	Distinguishes whether the evidence primarily evaluates model performance, the uncertainty output itself, or both; used to prevent evidence consisting only of accuracy or task performance from being treated as uncertainty evidence.
Validation_Protocol	Records the experiment, data split, or comparison procedure through which the evidence was obtained; used to determine whether the evidence constitutes internal evaluation, validation under external conditions, field validation, or comparison with a reference method.
AI_Uncertainty_Entry_Point	Locates where uncertainty is formed or enters the AI workflow, such as the output distribution, posterior approximation, sampling or ensemble, proxy score, or calibration interface; used to explain why similar outputs may have different generation mechanisms.
Assumption_Explicitness	Records whether assumptions related to the probabilistic model, data protocol, sensor, or physical process are stated explicitly; used to define the premises on which output interpretation depends and the extent to which they can be reviewed.
Physical_Information_Linked_To_Claim	Records the physical, sensing, measurement, or data-generation information explicitly linked to the dominant claim; used to distinguish real-world information that actually participates in constructing the claim from information that appears only as application background.
Physical_Condition_For_Claim	Records the data-generation, physical, measurement, or operational conditions on which the uncertainty claim depends; used to identify the applicability boundaries to which subsequent evidence must correspond.
Physical_Condition_Tested	Records how or whether claim-related conditions entered empirical evaluation, including direct testing, indirect testing, mention without testing, non-testing, and non-applicable states.
Physical_Trigger_Type	Records the specific trigger used to induce a condition change or stress test, such as noise, drift, device change, spatiotemporal extrapolation, or a dynamic event; used to characterize the scope of testing and its physical specificity.
Uncertainty_Manifestation	Records the identifiable response, robust behavior, or specific failure shown by the uncertainty output after testing or triggering; used as the evidential endpoint for determining whether uncertainty was actually evaluated.
Implementation_Channel_Change	Records whether implementation processes such as sampling, approximation, compression, proxy scoring, or post-hoc calibration changed the path through which uncertainty was represented; used to explain how a theoretical construction was converted into an operational output.
Computability_Motivation	Records the computational motivation for using the corresponding implementation or approximation scheme, such as latency, memory, dimensionality, numerical stability, or tractability; used to distinguish engineering-feasibility choices from evidence strength.
Measurement_Interpretation_Level	Records the level at which the output was interpreted—as an algorithmic score, statistical predictive uncertainty, measurement-result-related uncertainty, deployment reliability, or formal measurement uncertainty; used to control the boundary across which measurement meaning is extended.

**Table 2:** Coding distributions of uncertainty outputs, claim objects, and formation mechanisms (n = 556).

Coding Dimension	Category Distribution, Number of Studies (Percentage)
Uncertainty Output Type	Prediction interval/set/bounds: 137 (24.6%); Other/not explicit: 126 (22.7%); Predictive variance/dispersion: 92 (16.5%); Predictive confidence/probability: 64 (11.5%); Measurement result uncertainty: 13 (2.3%); other explicitly identified output types: 124 (22.3%)
Uncertainty Claim Object	Prediction reliability/confidence: 190 (34.2%); Model/epistemic uncertainty: 102 (18.3%); Calibration/coverage reliability: 92 (16.5%); Measurement result uncertainty: 35 (6.3%); Deployment/decision risk and Physical/parameter uncertainty: 18 (3.2%); other claim objects: 119 (21.4%)
AI Uncertainty Entry Point	Multiple uncertainty entry points: 343 (61.7%); Uncertainty proxy/score: 49 (8.8%); Computational approximation/sampling: 44 (7.9%); Predictive likelihood/output distribution: 30 (5.4%); Post-hoc mapping, calibration, or conformal interface: 30 (5.4%); other entry mechanisms: 60 (10.8%)
Assumption Explicitness	Not codable: 178 (32.0%); Explicit likelihood/prior: 151 (27.2%); Mixed/multiple assumptions: 103 (18.5%); Explicit sensor/physical model: 36 (6.5%); other assumption categories: 88 (15.8%)

Note: All percentages use the 556 codable studies as the denominator. The aggregated categories in the table are used only to condense the main-text table; their composition and all original enumerated categories are provided in the “Coding Dictionary” worksheet, and the study-level values are provided in the “Study Coding Matrix” worksheet of Supplementary File S1. Categories beginning with “Multiple” indicate that two or more relevant categories were identified within the same study; therefore, the proportion of a single category does not represent the overall occurrence of that feature.

**Table 3:** Coding distributions of physical conditions, validation methods, and uncertainty manifestations (n = 556).

Coding Dimension	Category Distribution, Number of Studies (Percentage)
Physical Information Linked to the Claim	Multiple physical information types: 332 (59.7%); Not identifiable/other: 65 (11.7%); Spatial/geometric structure: 47 (8.5%); Sensor/data-generation mechanism: 35 (6.3%); other single physical-information categories: 77 (13.8%)
Physical Condition for the Claim	Multiple physical conditions: 193 (34.7%); No identifiable physical condition: 155 (27.9%); Material/structural/load condition: 46 (8.3%); Spatial/environmental condition: 37 (6.7%); Support/domain/data-coverage condition: 29 (5.2%); Noise/SNR/artifact condition: 28 (5.0%); other single-condition categories: 68 (12.2%)
Physical Condition Tested	Indirectly tested: 256 (46.0%); Directly tested: 232 (41.7%); Not applicable: 31 (5.6%); Mentioned but not tested: 20 (3.6%); Not tested: 17 (3.1%)
Validation Protocol	Multiple validation protocols: 334 (60.1%); Not reported/not codable: 71 (12.8%); Random holdout/static split: 51 (9.2%); Stress/OOD/perturbation test: 36 (6.5%); Cross-validation/resampling: 21 (3.8%); Cross-domain/device/time/space validation: 18 (3.2%); other validation protocols: 25 (4.5%)
Physical Trigger Type	Not tested/not reported: 177 (31.8%); Multiple physical triggers: 117 (21.0%); Unknown category/unseen class/OOD: 109 (19.6%); Noise enhancement/SNR drop: 77 (13.8%); Cross-operating condition: 54 (9.7%); more specific physical triggers: 22 (4.0%)
Uncertainty Manifestation	Not reported/not codable: 251 (45.1%); Robust/trigger-responsive uncertainty: 184 (33.1%); Multiple uncertainty manifestations: 62 (11.2%); specific failure manifestations: 52 (9.4%); Not uncertainty-specific: 7 (1.3%)

Note: All percentages use the 556 codable studies as the denominator. “More specific physical triggers” includes Extreme/tail condition, Physical parameter/load variation, Transient/dynamic event, Cross-space/spatial extrapolation, Cross-device/instrument, and Cross-time/process drift. “Specific failure manifestations” includes Calibration degradation, Overconfidence under shift, Under-coverage, OOD detection failure, Variance non-response, and Interval non-expansion/collapse. The complete composition of the other aggregated categories is provided in the “Coding Dictionary” worksheet of Supplementary File S1.

**Table 4:** Unidimensional coding evidence supporting the existence judgment for the physical fidelity gap (n = 556).

Evidence-Chain Link (Coding Dimension)	Unidimensional Coding Observation	Role in the PFG Existence Judgment
Real-World Physical Linkage (Physical_Information_Linked_To_Claim)	In 491 studies (88.3%), physical or data-generation information linked to the claim could be identified.	Establishes a real-world referent for the PFG discussion; this item in itself does not indicate a gap.
Claim–Condition Node (Physical_Condition_For_Claim)	In 155 studies (27.9%), no identifiable claim-related condition was found.	An untraceable state occurs at the claim–applicable-condition node.
Condition–Test Node (Physical_Condition_Tested)	In 20 studies (3.6%), a relevant condition was mentioned but not tested; in 17 (3.1%), it was explicitly not tested; 31 (5.6%) were classified as Not applicable.	The first two categories directly show a breakpoint at which the condition did not enter empirical testing; not applicable is not treated as evidence of a gap.
Test–Response Node (Uncertainty_Manifestation)	In 251 studies (45.1%), the uncertainty manifestation was not reported or not codable; 7 (1.3%) reported only a manifestation that was not uncertainty-specific.	Shows missing or non-specific verifiable endpoints from empirical testing to the uncertainty output response.

Note: Each row is an independent unidimensional coding marginal distribution with 556 studies as the denominator. The proportions cannot be added and do not represent the same group of studies. The node states in the table are observations of PFG components, not PFG determinations for individual studies; this table does not estimate the proportion of studies involving PFG or judge its degree. Cross-field joint relationships and evidence-traceability stratification are presented in Section 4.

**Table 5:** PFG evidence-traceability questions and tier-assignment rules.

Question	Combined Coding Dimensions	Criterion for “Yes”	Evidence-Traceability Tier Outcome
Q1	Physical Condition for the Claim + Physical Condition Tested	An identifiable data-generation, physical, measurement, or operational condition entered testing directly or indirectly	No → Weak; Yes → Q2
Q2	Evidence Target + Uncertainty Manifestation	The evidence target includes uncertainty evaluation, and a codable uncertainty manifestation is reported	No → Weak; Yes → Q3
Q3	Physical Trigger Type + Validation Protocol	Additional trigger breadth, or cross-environment, field, or reference validation, is present	No → Medium; Yes → Strong

**Table 6:** Distribution of PFG evidence-traceability tiers across the 556 studies.

Evidence-Traceability Tier	Number of Studies	Percentage of the 556 Studies
Weak	347	62.4%
Medium	133	23.9%
Strong	76	13.7%

## Data Availability

The data supporting this review are provided in Supplementary File S1. The English-language workbook contains the complete 15-dimensional study coding matrix, 18,843 row-level coding evidence records, the coding dictionary, bibliographic information for the 566 included studies, item-level manual verification records showing the original and verified values, and study dimension coding adjudications. Evidence excerpts and locations are provided to support traceability to the source publications, which remain available from their respective publishers or databases. Intermediate searchable Markdown representations used during processing are not redistributed because they were derived from the source publications.

## Supplementary Materials

The following supporting information can be downloaded at: https://www.mdpi.com/article/10.3390/s26175447/s1, Supplementary File S1: Study coding and evidence verification, is provided with this manuscript as an English-language workbook. It contains seven worksheets: Instructions, Study Coding Matrix, Coding Evidence Chain, Coding Dictionary, Study Bibliographic Information, Manual Verification, and Coding Adjudication Summary.

## Abbreviations

The following abbreviations are used in this manuscript:

AI	Artificial intelligence
AUC	Area under the receiver operating characteristic curve
GUM	Guide to the Expression of Uncertainty in Measurement
M + S	Medium + Strong evidence-traceability tiers
ML	Machine learning
OOD	Out-of-distribution
PFG	Physical fidelity gap
PRISMA	Preferred Reporting Items for Systematic Reviews and Meta-Analyses
SAR	Synthetic aperture radar
SNR	Signal-to-noise ratio
UQ	Uncertainty quantification
URREF	Uncertainty Representation and Reasoning Evaluation Framework
